# Metabolic Adaptation in Heart Failure and the Role of Ketone Bodies as Biomarkers

**DOI:** 10.1007/s11897-024-00678-6

**Published:** 2024-09-07

**Authors:** Michael W. Foster, Joshua M. Riley, Praneet C. Kaki, Amine Al Soueidy, Ehson Aligholiazadeh, J. Eduardo Rame

**Affiliations:** 1 Department of Medicine, Division of Cardiology, Thomas Jefferson University Hospital, 833 Chestnut Street, Suite 600, Philadelphia, PA 19107 USA; 2 Sidney Kimmel Medical College, Thomas Jefferson University Hospital, Philadelphia, PA USA; 3 Department of Medicine, Copper University Hospital, Camden, NJ USA

**Keywords:** Heart failure, Cardiomyopathy, Biomarkers, Ketone bodies, Myocardial metabolism

## Abstract

**Purpose of Review:**

The development and progression of heart failure is characterized by metabolic and physiologic adaptations allowing patients to cope with cardiac insufficiency. This review explores the changes in metabolism in heart failure and the potential role of biomarkers, particularly ketone bodies, in staging and prognosticating heart failure progression.

**Recent Findings:**

Recent insights into myocardial metabolism shed light on the heart’s response to stress, highlighting the shift towards reliance on ketone bodies as an alternative fuel source. Elevated blood ketone levels have been shown to correlate with the severity of cardiac dysfunction, emphasizing their potential as prognostic indicators. Furthermore, studies exploring therapeutic interventions targeting specific metabolic pathways offer promise for improving outcomes in heart failure.

**Summary:**

Ketones have prognostic utility in heart failure, and potentially, an avenue for therapeutic intervention. Challenges remain in deciphering the optimal balance between metabolic support and exacerbating cardiac remodeling. Future research endeavors must address these complexities to advance personalized approaches in managing heart failure.

## Introduction

Heart failure is a complex, progressive syndrome that is marked by a multitude of adaptations allowing patients to live with cardiac insufficiency [1]. Adaptations in cardiac mechanics, hemodynamics, neurohumoral activation and response, and metabolism have been elucidated and form the basis for biomarker profiling and therapeutic targets [1–3]. Despite the formidable advances in this area of study, heart failure remains a progressive affliction claiming over 1 million hospitalizations in the United States alone with a very high mortality in the advanced stages [4].

Recently, advances in our understanding of myocardial metabolism have provided great insight into how the human heart adapts to physiologic challenges such as exercise and how the failing heart adapts to stress in the setting of cardiac insufficiency [3]. The failing myocardium is best understood as “an engine out of fuel” desperately trying to adapt to a bioenergetic deficit through metabolic pathways working on multiple levels of fuel production and energy transmission to sustain the cardiac workload of the mechanically disadvantaged failing heart [5]. In the context of this metabolic paradigm, one can understand how the reversal of heart failure can be achieved by “resting” the myocardium, decreasing heart rate, and improving mechanical efficiency through hemodynamic coupling and cardiac resynchronization [6–9]. Conversely, using the same paradigm of metabolic adaptation to energetic failure, it is clear why the use of contractility-enhancing therapies such as inotropes is detrimental and will accelerate disease progression increasing mortality in heart failure [10–12].

In this review, we will summarize the evidence for using metabolic biomarkers to stage and prognosticate disease progression in heart failure. For background, we will summarize the metabolic adaptations alluded to above and focus on the evidence for endogenous ketone production (ketogenesis) and ketone utilization which has recently been discovered as a metabolic response to fuel deprivation. We identify opportunities for future investigation, encouraging research teams to discover the translational potential of ketones as biomarkers and therapeutic targets in human heart failure.

## Role of Ketones in Myocardial Metabolism

The heart is an energy-intensive organ tasked with pumping thousands of liters of blood per day against pulmonary and systemic vascular resistance [13]. Carbohydrates and fatty acids are the primary metabolic substrates used by the heart to fulfill its steep energetic demands [3]. The heart can adapt to use other molecular sources of fuel based on varying conditions of substrate availability, including lactate, amino acids, and ketone bodies [14]. Under normal conditions, the heart does not significantly metabolize ketones due to the relative unavailability of ketones in a fed, physiological state [15]. Cardiac ketone utilization is typically limited to fasting or starvation conditions when glucose availability is low and ketones are readily formed [16].

Ketogenesis occurs in liver hepatocytes from fatty acids, which undergo β-oxidation to form acetyl-CoA, which is then converted to acetoacetate and β-hydroxybutyrate (BHB) through a series of intermediate enzyme-catalyzed reactions [17]. Ketone bodies are metabolized to produce acetyl-coenzyme A (CoA), the driver of the tricarboxylic acid cycle (TCA) [18]. Although glucose is more metabolically efficient than ketones due to its greater phosphate-to-oxygen (P/O) ratio, ketones are more efficient than fatty acids (FA), the primary fuel used by the myocardium. FA oxidation presents additional inefficiencies, namely increased mitochondrial uncoupling proteins and reactive oxygen species (ROS), which can diminish the ability of mitochondria to generate ATP [19]. The brain heavily relies on glucose for its energy supply [16]. Cardiac metabolism of ketones during fasting reduces the need for breaking down protein to fuel gluconeogenesis, preserving glucose for the brain [16]. Ketone metabolism is augmented in the heart when its capabilities for fatty acid and glucose oxidation are diminished as seen in the cardiac stress of advanced heart failure [20, 21].

There is evidence that sole ketone utilization is associated with myocardial complications. In a rat heart model, where ketones were the only metabolite available, mean cardiac output, diastolic, and systolic pressures were significantly reduced as compared to cardiac function with glucose present for utilization [22, 23]. Results revealed an accumulation of acetyl-CoA, citrate, and 2-oxoglutarate, indicating an inhibition of the TCA cycle at 2-oxoglutarate dehydrogenase [24]. Furthermore, this inhibition of 2-oxoglutarate dehydrogenase is associated with a drop in intramitochondrial concentrations of CoA. Since CoA is a required input in the reaction catalyzed by 2-oxoglutarate dehydrogenase, its sequestration effectively depletes TCA cycle intermediates, highlighting a key inefficiency of using ketone bodies as a fuel source [25]. When pyruvate, a glucose metabolite that forms TCA cycle intermediates, is present with acetoacetate, cardiac function and efficiency of the rat models no longer exhibit the decline observed during sole ketone utilization [26]. Evidently, a need exists for TCA cycle in optimal myocardial function.

There is conflicting evidence that ketones offer cardioprotective effects, notably in conditions of cardiac stress. Transgenic mice overexpressing D-β-hydroxybutyrate dehydrogenase 1, a key enzyme in ketone oxidation responsible for the interconversion of acetoacetate and β-hydroxybutyrate, demonstrated reduced ROS-mediated DNA damage and mitigated cardiac dysfunction when subjected to pressure overload-induced heart failure by aortic constriction [27]. Conversely, mice lacking succinyl-CoA:3-oxoacid CoA transferase, a key enzyme involved in ketone production, exhibited increased ROS activity in the myocardium, increased left ventricular volume, and diminished ejection fraction compared to control mice when subjected to pressure overload by aortic constriction. [28].

## Metabolism in Heart Failure

Cardiac metabolism comprises three main components: substrate utilization, oxidative phosphorylation, and transport of energy (via ATP) to the myofibrils through the creatine kinase energy shuttle [13, 29]. In heart failure, all three of these components of metabolism are affected [29]. There is a reduction in substrate uptake, as fatty acid utilization is decreased in advanced heart failure, with rate of decrease correlating with progression of cardiac disease [30]. Glucose utilization is also decreased in cardiac failure, due to worsening insulin resistance in the myocardium and reduced expression of key factors involved in the transport and metabolism of pyruvate [30]. Oxidative phosphorylation is impaired due to cardiac mitochondrial abnormalities, resulting in decreased energy production [5, 29]. There is impairment in ATP synthase activity, as well as in electron transport chain complexes. When compared to normal cardiac function, ATP production can be reduced by at least 30% in advanced heart failure [5]. The level of uncoupling proteins is increased, which causes significant energy to be lost through heat rather than ATP production in the mitochondria [31].

Transport of energy to the cardiac myofibrils is also affected in heart failure. The decline in ATP transfer and utilization is due to a combination of decreased creatine kinase activity and depletion of high-energy phosphates [32]. This causes a decrease in energy delivery to the myofibrils by up to 71%, leading to loss of contractile function and loss of inotropic reserve with clinical manifestation of dyspnea on exertion [5]. Both the mitochondrial and myofibrillar creatine kinase activities are affected in heart failure [32]. Mitochondrial creatine kinase catalyzes the transfer of energy of high-energy phosphate from ATP to creatine, facilitating storage of energy in the form of phosphocreatine. Phosphocreatine can easily diffuse from mitochondria to myofibrils, where it is transformed back to ATP via the myofibrillar creatine kinase, but the failing heart displays lower levels of phosphocreatine than the normal heart [14, 33]. The assessment of cardiac energy metabolism is done using phosphorus-31 magnetic resonance spectroscopy that can detect levels of ATP and phosphocreatine [34]. Myocardial phosphocreatine/ATP ratio is reduced in cardiac failure and correlates with diastolic dysfunction, systolic dysfunction, and New York Heart Association classes [34].

At a molecular and genetic level, there are factors that affect the expression of genes that encode molecular regulators for energy metabolism. One of the main nuclear-receptor transcription factors is the peroxisome proliferator–activated receptor α (PPARα) that controls fatty acid oxidation. PPARα is reduced in heart failure, shifting cardiac energy utilization from fatty acid to glucose [35]. The deletion of PPARα gene resulted in decreased contractile reserve and depleted cardiac energy stores during inotropic challenge [36].

These intricate alterations in metabolism have been further studied to elucidate the role of various molecules in energy production and utilization during states of cardiac dysfunction. A study conducted by Bedi et al. (2016) aimed to further characterize the fatty acid utilization and carbohydrate metabolism in patients with non-ischemic heart failure. The results from this study indicate a reduction in concentrations of lipid intermediates, and instead a reliance on ketone utilization to fuel the failing heart [30]. This conclusion aligns with the above-discussed components of the compromised cardiac energy metabolism pathway in heart failure. More specifically, the reliance on ketone bodies as a source of energy stems from observed findings in heart failure regarding a decrease in fatty acid and glucose utilization, ATP synthase and respiratory chain complexes activities, PPARα, ATP synthase, phosphocreatine, creatine kinase activity, and ATP transfer [29–31, 34, 36].

Understanding how the three main components of energy metabolism are affected in patients with heart failure may have clinical and therapeutic implications, and metabolic therapy is a promising treatment of patients with heart failure: by modulation of substrate utilization, stimulation of oxidative phosphorylation, or manipulation of high-energy phosphate metabolites. Future studies are needed to explore possible therapeutic interventions to improve cardiac energy metabolism.

## Evidence for Metabolic Biomarkers in Heart Failure

Metabolic biomarkers are of interest with heart failure as they can provide non-invasive data for both diagnosis and future prognosis. These can allow for earlier detection of the disease with the goal of improved morbidity and mortality. The main metabolic markers under study include nitric oxide, arginine, long-chain acylcarnitines, pentane, and ketones.

A study performed in 2011 by Janardhan et al. analyzed alterations in ketone body metabolism in advanced heart failure [37]. The study population consisted of 11 patients with advanced heart failure and 10 patients without heart failure undergoing electrophysiologic procedures. They evaluated ketone levels in both myocardial and skeletal muscle cells. Findings from the study indicate there is tissue-specific alteration in ketone utilization. There was noted impairment in ketone body metabolism in skeletal muscle cells. They posed the question of whether this dynamic alteration can be used as a biomarker for skeletal myopathy and progression of heart failure [37].

To further investigate this topic, Ho et al. (2019) utilized cardiac models in mice to study the contribution of this substrate in cardiac energy production and subsequent contractile efficiency [15]. Palmitate, glucose, and increasing concentrations of β-hydroxybutyrate were introduced to the cardiac models. Although ketones become the key source of energy for the failing heart, the increase in ketogenesis does not result in significant increase in cardiac work [15]. Thus, there was no increase in cardiac efficiency with sole reliance on ketones.

The reliance on ketone bodies as fuel, and the above conclusion that this substrate does not improve cardiac efficiency, points to a possible relationship between ketosis and heart failure progression. In a study examining the trend of blood ketones in 45 patients with chronic heart failure compared to 14 individuals without cardiac dysfunction, there were significantly elevated blood ketone bodies in heart failure (median 267 µmol/L) compared to normal subjects (median 150 µmol/L). There was found to be an association between ketone bodies and pulmonary artery wedge pressures, left ventricular ejection fraction, right atrial pressure, and circulating free fatty acids [20]. It was discovered that this metabolite was elevated in heart failure and that the degree of elevation was proportional to the severity of cardiac dysfunction [20]. These findings were re-demonstrated by Aubert et al. (2017); quantitative mitochondrial proteomics were used to study metabolic derangements in mouse models for heart failure [21]. Compared to the control group, the mice in the heart failure group had higher concentrations of ketone body utilization. This study demonstrated the reliance on this form of energy when cardiac function is compromised and the heart begins undergoing cardiac remodeling [21].

These studies help demonstrate the role of ketone bodies in the failing heart. As cardiac function worsens in the setting of pressure or volume overload, there is a reliance on generation of ketones as alternative fuel source. Unfortunately, this source of energy is not ideal, and it does not bode well for cardiac efficiency. Although not the most efficient pathway for the heart, the lack of ketone body generation at the same time can result in quicker onset of heart failure. Because of this intricate relationship, ketone bodies may potentially serve as metabolic biomarkers for heart failure. The degree of elevation in this substrate may be a marker of worsening cardiac function.

Regarding possible therapeutic approaches and targets, a study investigated TCA cycle intermediate levels in the failing hearts of mice. Investigators found not only that succinyl CoA levels were lower in the hearts of mice with ischemic cardiomyopathy, but dietary addition of 5-aminolevulinic acid can restore succinyl CoA levels and was shown to provide beneficial cardiac reverse remodeling [38]. Exercise tolerance and objective cardiac systolic function was improved with this dietary addition. It is reasonable to conclude that addition of dietary supplementation to the altered biochemistry of individuals with heart failure is a promising treatment avenue yet to be fully explored. But what about the ketones that the human body forms naturally?

Endogenous ketones, namely BHB that is formed in abundance during the very-low-carbohydrate intake diet (ketogenic diet [KD]), provide a different avenue for exploration with regards to heart failure treatment. Limitations to this form of treatment exist, including dietary adherence, increased LDL and triglyceride levels, transient endothelial dysfunction, and risk of lipotoxicity hinder KD as a viable, long-term, durable treatment for heart failure [39–41]. Additionally, the lipolysis that is characteristic of KD may worsen myocardial perfusion, which would be especially harmful for patients with ischemic cardiomyopathy or underlying microvascular dysfunction [41].

Exogenous ketones, supplemented to highlight the metabolic changes of heart failure, has yielded another area of scientific progress in this field. A small study analyzed cardiovascular changes in 16 patients admitted with HFrEF were administered an intravenous exogenous ketone ester, 3-hydroxybutyrate (3-OHB) or placebo. Increases in heart rate, stroke volume, and cardiac output were observed in the patients who received 3-OHB infusion [42]. Another study, recently published in *Circulation*, investigated dietary addition of an exogenous ketone ester, 3-hydroxybutyrate (3-OHB), in patients with chronic ambulatory heart failure with reduced ejection fraction (HFrEF). Subjects were given a 3-OHB supplement or isocaloric placebo drink with subsequent washout period, then sequence-controlled switched between treatment arms. It was found that 3-OHB supplementation resulted in mildly increased left ventricular ejection fraction, increased cardiac output, decreased ventricular filling pressure, and decreased NT pro-BNP levels when compared to the same subjects after placebo supplementation [43]. These new findings underscore the importance of understanding the role of ketone supplementation in HFrEF given that we have growing evidence of reverse remodeling among patients with chronic heart failure.

The role of sodium-glucose contransporter-1 and sodium-glucose cotransporter-2 (SGLT2) inhibitors provides another area for future study in the field of ketone metabolism in heart failure. There has been consistent data to support increased ketogenesis with multiple of the SGLT2-i’s; however, whether this increased ketosis is partially responsible for the reverse remodeling and mortality benefit seen with SGLT-2i use in patients with heart failure is yet to be elucidated [44–47].

## Conclusion

Metabolic adaptations are critical in the ability of the human heart to provide a cardiac output, commensurate with the needs of the body under conditions of physiologic and pathologic stress. We know that the mammalian heart is capable of switching from utilization of one substrate to another, depending on conditions of physiologic demand with exercise, fasting, and abnormal management of fuel access. The failing human heart can rely on ketones in order to adapt to the known bioenergetic deficit in heart failure [30]. It is thus not surprising that ketone pathway elements can be invoked to understand, risk stratify, and treat cardiovascular disease.

Recent research has shown that acute nutritional ketosis via dietary supplementation of ketone esters can improve exercise tolerance and cardiac performance in performance athletes [48]. Given the reliance on ketone bodies for the failing heart, perhaps nutritional ketosis in patients with heart failure would provide necessary substrate to support the altered metabolism.

Or would it further the downward spiral of cardiac remodeling?

Research is needed to answer these questions and improve outcomes as heart failure is a devastating syndrome for patients, families, and society.

## Data Availability

No datasets were generated or analysed during the current study.
